# Effects of Strength Training on Body Composition in Young Male Professional Soccer Players

**DOI:** 10.3390/sports7050104

**Published:** 2019-05-05

**Authors:** Luis Suarez-Arrones, Pilar Lara-Lopez, Nacho Torreno, Eduardo Saez de Villarreal, Valter Di Salvo, Alberto Mendez-Villanueva

**Affiliations:** 1 Performance Department, FC Basel 1893, 4002 Basel, Switzerland; pilarlaralopez74@gmail.com (P.L.-L.); nachotorreno@hotmail.com (N.T.); 2 Department of Football & Science, Pablo de Olavide University, 41013 Sevilla, Spain; esaesae@upo.es; 3 Football Performance & Science Department, ASPIRE Academy, 22287 Doha, Qatar; valter.disalvo@aspire.qa (V.D.S.); amendezvillanueva@yahoo.com (A.M.-V.); 4 Department of Movement, Human and Health Sciences, University of Rome “Foro Italico”, 00135 Roma, Italy

**Keywords:** soccer, eccentric overload, neuromuscular training, lean mass, DXA

## Abstract

The present prospective cohort study investigated changes in body composition (BC) in young male football players (*n* = 18, 16.1 ± 0.8 years; 181.0 ± 0.1 cm; 71.3 ± 4.9 kg) after combined football and strength training (ST) during a whole in-season period (26 weeks). BC was measured at whole-body absolute and regional levels by dual-energy X-ray absorptiometry in eighteen players at the beginning and at the end of the competitive period. The ST was organized into three different session types: ST in the gym, specific ST on the field, and individual ST (weak points). The results of the present study indicated that fat-free mass (FFM) was substantially higher following the competitive period (5.1% ± 1.2%), while percentage of fat showed no changes during the competitive period. At the regional level, arms’ and legs’ FFM increased at the end of the season, and bone mineral content (BMC) and density (BMD) increased in arms, legs, pelvis, thoracic spine, and lumbar spine. In conclusion, within the limitation of the potential positive impact of growth and/or maturation, present results seem to indicate that an ST program that supplements football-related training sessions could be an effective option to increase FFM, BMC, and BMD at both whole-body and regional level across the competitive season in young male professional football players.

## 1. Introduction

The central goal of strength training (ST) in football is to improve the players’ specific activities inherent to the sport [1], as well as to reduce post-training and post-match markers of muscle damage [2] and minimize the risk of injury [3]. Football is considered a high-intensity intermittent sport requiring high levels of physical fitness related to the ability to perform powerful actions [4], with a varied range of activities that involve both breaking and propulsive forces as well as distinct contraction modes and velocities [1]. In addition, a professional football player can perform about 50 turns during an official game [5]. While the predominant activity patterns during match-play are aerobic in nature, the biggest determinant factors of game outcomes depend on anaerobic mechanisms [6]. 

Many different methods to improve strength and power have been suggested throughout the years. Certain ST methods in football combine different exercise modes, such as weight training and ballistic exercises, plyometric training, and sports-specific force-based actions. A training method that has increased in popularity in the past two decades is flywheel training, especially among professional football players [7,8,9,10]. With these devices, the load is generated by the inertia of a rotating mass (flywheel) providing an inertial resistance during coupled concentric and eccentric actions, and flywheel exercises can, if properly used, produce greater eccentric overload and muscle activation [7,8]. A recent meta-analysis provided evidence supporting the benefits of flywheel ST to promote skeletal muscle adaptations expressed as strength, power, and muscle size [11]. Only recently did a study describe the changes in body composition (BC), strength, and sprint performance in response to an entire competitive season of football and ST using flywheel devices in professional football players [10]. Furthermore, to date, no study has assessed the effect of regular flywheel ST on BC in young male football players for a whole season.

BC is a key fitness element relevant to football player performance [12], and in professional football, it is traditionally evaluated several times throughout the season to monitor the efficacy of training and nutrition. In addition, non-optimal BC may adversely influence football performance and the risk of injury [12]. As an example, excessive fat mass will load the football player with extra body weight, potentially affecting power output and demanding greater energy expenditure during the game [13]. On the other hand, lean muscle mass can positively impact power performance in team-sport players [14]. The BC of young people undergoes rapid changes during their growth spurts, with substantial changes in height and weight [15]. The height and weight development of young football players during puberty is similar to that of the general population, and the only difference found in BC is that football players tend to be leaner than average young people [16]. Reilly et al. [17] showed that elite young male football players were significantly leaner, possessed more aerobic power, and were more tolerant to fatigue than sub-elite football players. In addition, the best longitudinal predictor for leg power in late-adolescence football players was fat-free mass [18]. Also interesting is the fact that young soccer players show a high percentage of body fat due to absolute low levels of lean mass and not high levels of fat mass per se [18]. To the author’s knowledge, no previous investigation has assessed the effects of a combined football and neuromuscular training program in BC, using DXA (dual-energy X-ray absorptiometry), in young professional football players. For this purpose, the aim of the present investigation was to assess the variations in BC in response to an entire competitive season of football-related training supplemented with an eccentric-biased ST program in young male professional football players.

## 2. Materials and Methods

### 2.1. Experimental Design

The present prospective observational cohort study employed controlled repeated measures to analyze in-season BC changes after a combined football and ST program for 26 weeks in young male professional football players. Throughout the season, players usually had five training sessions (mean duration, 65 min), plus the ST program. BC was assessed using DXA in September (at the beginning of the in-season period) and in June (at the end of the season). Players did not perform any heavy exercise 48 h before the BC assessment.

### 2.2. Participants

Data were collected from a group of male young football players belonging to the third team from a professional football club (playing in Europa League). Players spent ~7 h in training plus one official match per week, and the team competed at the highest level for this age group. Our data came from routine testing over the season; therefore, ethics committee clearance was not required. The study conformed, nevertheless, with the current national and international laws and regulations governing the use of human subjects (Declaration of Helsinki II) and was approved by the Ethics Committee of the involved institutions (Anti-Doping Lab Qatar Institutional Review Board, IRB number: E2013000004). Written consent was obtained from the participants before the start of the study, or their legal representatives if the player was younger than 18 years old, after being fully informed about the experimental procedures, purpose, and potential risks. Only the data from players who completed >85% of the strength training sessions were included (*n* = 18, 16.1 ± 0.8 years; 181.0 ± 0.1 cm; 71.3 ± 4.9 kg).

### 2.3. Measures

#### Body Composition Examination

Body mass was measured with an electronic scale (OHAUS Corp., Florham Park, NJ, USA) and stature with a stadiometer (Seca 213, Hamburg, Germany). Body composition (fat-free mass (FFM), fat mass (FM), bone mineral content (BMC), and bone mineral density (BMD)) were assessed by DXA (Hologic QDR Series, Delphi A model, Bedford, MA, USA) using Hologic APEX software (Version 13.3:3, Hologic, Bedford, MA, USA), and according to the manufacturer’s recommended procedures. Participants were presented in a rested, fasted, and hydrated state and were instructed to avoid strenuous exercise for 24 h prior to testing. Subjects wore only shorts and removed any metal and jewelry prior to assessment. Before any measurements, the DXA was calibrated each day with phantoms, as per the manufacturer’s guidelines. The participants assumed a stationary, supine position on the scanning table, with hands level with the hips and feet slightly apart, as in a recent study [10]. All scanning and analyses were performed by the same operative to certify reliability and in accordance with standardized testing protocols recognized as best practice [18,19,20]. Whole-body data are reported as total body, excluding the head [21]. BC by DXA was measured at the same time in the morning in September (at the start of the in-season period) and in mid-June (at the end of the season).

### 2.4. Training

Subjects participated in a weekly football training program with one day of rest (Monday), and the players were involved in an official game once a week (typically on Sunday). The entire squad complemented the football training with an ST program for almost the entire in-season period (26 weeks). The complete ST program was organized into three different session types: (i) ST in the gym; (ii) specific ST on the field; and (iii) individual training.

ST in the gym was usually organized as circuit training before the football drills in the field. Players performed one or two laps of a circuit consisting of 10–12 exercises mainly focusing on the lower limbs, combining free weights with non-gravity dependent flywheel inertial devices (Kbox® and Versa-Pulley®), and including some functional exercises for upper-body and core muscles. In addition, complementary ST sessions were prescribed with exercises for upper-body, core, and lumbopelvic stability. ST sessions in the gym lasted 25–35 min each, while complementary sessions lasted ~20 min. Specific ST on the field lasted 20–25 min each and consisted of different combined football drills with goal-shooting (finishing), including high-intensity actions such as plyometric jumps, resisted sprint, duels, different change of directions, or high-speed running, among others. Individual training consisted of ST sessions in the gym focusing on the player’s weak points for injury prevention, and these sessions were usually planned after the football training on the field and lasted 10–15 min. During the 26 weeks of ST, only data from players who completed >85% of the ST sessions in the gym and on the field were included. In addition to the 26 weeks of ST, the players did not perform the complete ST program for ~9 weeks due to friendly games, international weeks, or because there was a reduction in training load. In those cases, players only completed the individual ST.

### 2.5. Statistical Analysis

Data in the text and tables are shown as means with standard deviations (SD) and 90% confidence limits. All data were first log-transformed to reduce bias arising from non-uniformity error. For players, individual differences in BC from the initial values were compared with the smallest worthwhile difference (SWD), which was set as 0.2 of the typical error of the estimate [22]. For individuals, longitudinal differences in DXA variables were evaluated using standardized differences, based on Cohen’s effect size (ES) principle. Threshold values for assessing magnitudes of the ES were >0.20, 0.20, 0.60, 1.2, and 2.0 for trivial, small, moderate, large, and very large, respectively [22]. Probabilities were used to make a qualitative probabilistic mechanistic inference about the true differences within-player, and changes were considered as substantial when the probabilities were >75% [23,24], which occurs when the difference is greater than the sum of the SWD and the typical error of measurement [22]. The scale for interpreting the probabilities was as follows: ≤1%, almost certainly not; >1–5%, very unlikely; >5–25%, unlikely; >25–75%, possible; >75–95%, likely; >95–99%, very likely; >99%, almost certainly [22].

## 3. Results

Players´ height showed no substantial changes during the in-season period (from 1.81 ± 0.05 to 1.82 ± 0.05 cm; ES = 0.15 ± 0.05 (6/94/0)).

### 3.1. Whole-Body Composition

BC variations after the in-season period are presented in Table 1. Body mass (BM) and FFM were almost certainly higher following the competitive period (4.7% ± 1.3% and 5.1% ± 1.2%, respectively). FM (%) showed no statistical changes.

### 3.2. Variations at Regional Levels

Variations in left and right arms after the in-season period are presented in Table 2. The mass, FFM, BMC, and BMD for arms almost certainly increased during the in-season period. A very likely reduction in arm FM (%) was only observed in left arms.

Variations in the left and right legs after the in-season period are presented in Table 3. The mass, FFM, BMC, and BMD for legs substantially increased during the in-season period. Left leg FM (%) was almost certainly reduced during the in-season period while there were unclear changes in right leg FM (%).

Variations in the trunk, pelvis, spine, and ribs after the in-season period are presented in Table 4. Trunk mass, pelvis BMC and BMD, thoracic spine BMC and BMD, lumbar spine BMC and BMD, and rib BMD were substantially increased during the in-season period. 

## 4. Discussion

The purpose of this study was to investigate the impact of combined football and ST on BC in young male professional football players. The major findings of the research were that (a) whole-body FFM increased after the competitive season; (b) at regional levels, lean muscle mass for arms and legs increased at the end of the season, as well as BMC and BMD in arms, legs, pelvis, thoracic spine, and lumbar spine.

Assessment of BC is regularly undertaken when players are being monitored during the regular season. Previous studies investigated BC changes during a full season [21,25] or only during the competition period [10] in adult professional football players. To our knowledge, no peer-reviewed research study has described the effects of combined football and ST on BC (DXA-derived measurements) in young male football players. The results of the present study showed no changes in FM during the in-season period, and FM (%) at regional levels only decreased in the arm and the left leg at end-season. With similar strength training methodology, DXA-derived measurements showed a whole-body reduction in absolute and relative %FM during the competitive period in senior professional football players [10]. The difference in playing one or two games a week in conjunction with the age of the players and the possible maturation process could have possibly influenced the results. Also using DXA, a decrease in FM was found by Milanese et al. [21] in adult elite football players during the in-season period in comparison to the pre-season values, although within the in-season period, an increase in lower limb and trunk FM was shown. Using skinfolds, similar results were reflected with adult elite football players from the French league [25]. In this study, a decrease in FM was found after the pre-season period, but FM did not change during mid-season, while a substantial increase was reflected at the end of the season, probably due to the tuning down of training intensity in the last weeks of the competitive season [25]. 

The results of the present study showed substantial whole-body increases in FFM (5.1%) after the in-season training period. A recent previous study with professional football players showed lower increments in FFM (2.5%) during the competitive period after an eccentric-overload ST program [10]. Different from our results, increases in FFM were described with adult elite football players during the competitive period in comparison to the pre-season, while no changes were shown within the in-season period [21]. Using skinfolds, similar results were shown with adult elite football players from the French League, with no changes in FFM within the competitive period [25]. These differences between previous studies [10,21,25] and the results of the present investigation are probably due to the different global training methodologies (football + neuromuscular training), age of the players, and possible maturation. In this regard, the lack of a control group does not allow us to draw firm conclusions into the direct link between the performed training program and the reported increases in FFM. However, taken alone, the observed increases in muscle mass in the present study seem particular interesting since for football players in late adolescence (i.e., after peak height velocity), as in the present study, the only longitudinal predictor for leg power was fat-free mass [26], suggesting that the gains in muscularity might have benefited the power in the current sample of players. 

Our results showed that when FFM was analyzed at regional levels, increases in FFM were identified in the arm and leg regions. Similar results were shown in the arms and trunk, but not in the leg FFM after a similar in-season combined football and eccentric-overload training with professional football players [10]. Increases in upper-body (arms + trunk) FFM were presented by Milanese et al. [21] during the in-season period, but only when compared to pre-season, and lower limb FFM remained unchanged over the season (pre-season and competitive period). A decrease in lean mass and muscle strength deficiency was proposed as one of the main risk factors associated with muscle strain injuries in football [3,8,23,27], and there is a relationship between the high force production capabilities of football players and the expression of reduced post-match markers of muscle damage [2]. Therefore, a greater lower limb muscular strength may reduce muscular damage following match-play and the muscle strain injury rate in football. Although we did not evaluate muscle strength, previous studies have shown that a neural factor accounts for the early strength gains occurring during the first weeks of training, while a hypertrophic factor has been claimed to have a later onset [28,29]. The relative increase in muscle strength is greater than what could be accounted for by the increase in muscle volume [30]. In elite football, the strength and control in both the upper body and core may influence performance by helping abilities such as sprinting, jump, agility, or change of direction, having a high impact in all types of duels and contact actions during match-play.

Previous research has indicated that football training is associated with positive changes in BMC and/or BMD [31,32]. Only two studies [10,21] examined changes in BMC and BMD through the season in professional football players. Milanese et al. [21] showed an increase in BMC and BMD in the pelvis and upper limbs during the in-season period, but with no changes in the lower limbs. By contrast, the results of the present study and Suarez-Arrones et al. [10] showed that BMC and BMD increased at all regional levels (arms, legs, pelvis, and thoracic spine) at end-season in comparison to the beginning of the competitive period. These findings suggest that the combined football and ST experience is beneficial to bone strength over a 9-month in-season period in young professional football players, also taking into account the differences in the age of the players and possible maturation process.

One of the limitations of the current research is that our study assessed young professional players from an elite professional football club, and this group of participants did not allow us to have a control group (due to professional ethics). Additionally, the influence of diet on BC was not exhaustively controlled throughout the season. Some players had breakfast, lunch, and dinner at the club (balanced diet for footballers), but there was not a daily diary of food intake. Other players were instructed to follow a similar balanced diet at home. Finally, the statistical approach used in the present study was magnitude-based inference. Several statisticians have criticized magnitude-based inference due to theoretical problems with the method [33]. 

## 5. Conclusions

Within the limitations of the absence of a control group and the potential positive impact of growth and/or maturation, the present results seem to indicate that an ST program that supplements football-related training sessions in young professional soccer players could be an effective option to increase FFM, BMC, and BMD at both whole-body and regional level through the competitive season. Furthermore, neuromuscular training may help players to reduce muscular damage following match-play, as shown in previous studies [2]; such training is beneficial to bone strength and remodeling throughout the competitive season in football. The combined training program was suitable for inducing optimal changes to BC parameters that are relevant in helping to maximize football performance in young football players. The results of the present study should be taken into consideration by the coaches when planning the training prescription and control of physical performance in young football players during the season. More research, including a control group, is necessary to verify these results.

## Figures and Tables

**Table 1:** Variations in fat-free mass (FFM), fat mass (FM), and body mass (BM) after an in-season training period. Data are mean ± SD.

Variables	Beginning of the In-Season Period	End of the Season	Standardized Differences & QA (90% CL)
Body Mass (kg)	66.8 ± 5.3	69.9 ± 5.0 ^a^	0.56 ± 0.16 (↑100/0/0)
Fat Mass (kg)	11.0 ± 1.6	11.3 ± 1.9	0.18 ± 0.29
Fat Mass (%)	16.5 ± 2.1	16.2 ± 2.6	−0.15 ± 0.26
Fat-Free Mass (kg)	55.8 ± 4.7	58.6 ± 4.6 ^a^	0.58 ± 0.14 (↑100/0/0)

QA = qualitative assessment; CL = confidence limits; ^a^ substantial difference vs. beginning of the in-season period.

**Table 2:** Variations in left and right arms after the in-season training period. Data are mean ± SD.

Variables	Beginning of the Competitive Period	End of the Season	Change in Mean (%)	Standardized Differences (90% CL)	Qualitative Assessment	
Left Arm Mass (g)	4076.9 ± 539.9	4603.6 ± 488.1	13.2 ± 4.1	0.93 ± 0.28	*Almost Certainly* *↑*	100/0/0
Left Arm Fat Mass (g)	659.5 ± 122.8	651.4 ± 131.1	−1.4 ± 6.8	−0.06 ± 0.34	*Unclear*	10/66/24
Left Arm Fat Mass (%)	16.2 ± 2.1	14.3 ± 3.8	−12.9 ± 6.6	−0.82 ± 0.48	*Very Likely* *↓*	0/2/98
Left Arm FFM (g)	3417.4 ± 459.7	3952.2 ± 507.2	15.6 ± 4.7	1.11 ± 0.32	*Almost Certainly* *↑*	100/0/0
Left Arm BMC (g)	161.6 ± 24.0	211.7 ± 27.5	31.3 ± 2.6	1.99 ± 0.17	*Almost Certainly* *↑*	100/0/0
Left Arm BMD (g∙cm^−2^)	0.74 ± 0.05	0.83 ± 0.08	11.7 ± 2.3	1.81 ± 0.43	*Almost Certainly* *↑*	100/0/0
Right Arm Mass (g)	4306.9 ± 544.4	4821.0 ± 406.0	12.4 ± 3.6	0.90 ± 0.28	*Almost Certainly* *↑*	100/0/0
Right Arm Fat Mass (g)	726.4 ± 191.9	853.8 ± 195.8	18.0 ± 7.8	0.63 ± 0.34	*Very Likely* *↑*	98/2/0
Right Arm Fat Mass (%)	16.7 ± 2.9	17.7 ± 3.6	5.0 ± 6.0	0.30 ± 0.36	*Possibly*	68/31/1
Right Arm FFM (g)	3580.5 ± 418.6	3967.2 ± 340.5	11.1 ± 3.5	0.88 ± 0.30	*Almost Certainly* *↑*	100/0/0
Right Arm BMC (g)	171.0 ± 22.7	213.7 ± 24.3	25.3 ± 2.7	1.80 ± 0.20	*Almost Certainly* *↑*	100/0/0
Right Arm BMD (g∙cm^−2^)	0.76 ± 0.05	0.85 ± 0.06	11.9 ± 1.3	1.68 ± 0.20	*Almost Certainly* *↑*	100/0/0

FFM = fat-free mass; BMC = bone mineral content; BMD = bone mineral density; CL = confidence limits.

**Table 3:** Variations in left and right legs after the in-season training period. Data are mean ± SD.

Variables	Beginning of the In-Season Period	End of the Season	Change in Mean (%)	Standardized Differences (90% CL)	Qualitative Assessment	
Left Leg Mass (g)	13,344.7 ± 1027.4	13,766.7 ± 1031.9	3.2 ± 1.8	0.39 ± 0.23	*Likely* *↑*	92/8/0
Left Leg Fat Mass (g)	2526.8 ± 376.8	2313.6 ± 446.7	−9.0 ± 6.4	−0.54 ± 0.37	*Likely* *↓*	0/6/93
Left Leg Fat Mass (%)	19.0 ± 2.6	16.8 ± 2.9	−11.8 ± 6.0	−0.82 ± 0.40	*Almost Certainly* *↓*	0/1/99
Left Leg FFM (g)	10,817.9 ± 926.1	11,453.1 ± 884.1	5.9 ± 2.2	0.66 ± 0.24	*Almost Certainly* *↑*	100/0/0
Left Leg BMC (g)	524.8 ± 69.7	620.3 ± 64.6	18.6 ± 2.5	1.31 ± 0.17	*Almost Certainly* *↑*	100/0/0
Left Leg BMD (g∙cm^−2^)	1.20 ± 0.12	1.36 ± 0.10	13.5 ± 2.8	1.23 ± 0.26	*Almost Certainly* *↑*	100/0/0
Right Leg Mass (g)	13,450.6 ± 926.3	14,230.6 ± 1058.0	5.8 ± 2.0	0.80 ± 0.28	*Almost Certainly* *↑*	100/0/0
Right Leg Fat Mass (g)	2515.8 ± 368.7	2676.3 ± 547.0	5.4 ± 5.9	0.42 ± 0.40	*Likely* *↑*	82/17/1
Right Leg Fat Mass (%)	18.7 ± 2.5	18.7 ± 3.4	−0.4 ± 4.8	0.02 ± 0.34	*Unclear*	19/67/14
Right Leg FFM (g)	10,934.9 ± 823.4	11,554.3 ± 901.1	5.6 ± 1.8	0.72 ± 0.24	*Almost Certainly* *↑*	100/0/0
Right Leg BMC (g)	523.8 ± 64.5	624.8 ± 79.4	19.2 ± 2.2	1.50 ± 0.21	*Almost Certainly* *↑*	100/0/0
Right Leg BMD (g∙cm^−2^)	1.22 ± 0.13	1.39 ± 0.13	13.6 ± 2.6	1.26 ± 0.26	*Almost Certainly* *↑*	100/0/0

FFM = fat-free mass; BMC = bone mineral content; BMD = bone mineral density; CL = confidence limits.

**Table 4:** Variations in trunk, pelvis, thoracic spine, lumbar spine, and lumbar rib after the in-season training period. Data are mean ± SD.

Variables	Beginning of the In-Season Period	End of the Season	Change in Mean (%)	Standardized Differences (90% CL)	Qualitative Assessment	
Trunk Mass (g)	31,619.6 ± 2797.8	32,500.4 ± 2530.9	2.9 ± 1.5	0.30 ± 0.16	*Likely* *↑*	86/14/0
Trunk Fat Mass (g)	4568.9 ± 671.7	4797.8 ± 788.4	4.8 ± 3.5	0.33 ± 0.24	*Likely* *↑*	81/19/0
Trunk Fat Mass (%)	14.5 ± 1.9	14.8 ± 2.3	1.9 ± 2.9	0.15 ± 0.22	*Unclear*	35/64/1
Trunk FFM (g)	27,050.7 ± 2527.5	27,702.6 ± 2349.9	2.5 ± 1.5	0.25 ± 0.15	*Possibly*	71/29/0
Pelvis BMC (g)	370.3 ± 54.9	470.5 ± 67.2	27.2 ± 3.2	1.74 ± 0.22	*Almost Certainly* *↑*	100/0/0
Pelvis BMD (g∙cm^−2^)	1.29 ± 0.12	1.57 ± 0.13	21.7 ± 1.2	2.24 ± 0.12	*Almost Certainly* *↑*	100/0/0
Thoracic Spine BMC (g)	72.3 ± 15.2	102.1 ± 16.2	42.4 ± 9.3	1.87 ± 0.49	*Almost Certainly* *↑*	100/0/0
Thoracic Spine BMD (g∙cm^−2^)	0.69 ± 0.08	0.85 ± 0.08	24.7 ± 4.3	1.92 ± 0.36	*Almost Certainly* *↑*	100/0/0
Lumbar Spine BMC (g)	64.4 ± 15.0	79.3 ± 16.4	24.1 ± 8.9	0.95 ± 0.41	*Almost Certainly* *↑*	100/0/0
Lumbar Spine BMD (g∙cm^−2^)	0.98 ± 0.12	1.17 ± 0.12	19.2 ± 2.7	1.54 ± 0.23	*Almost Certainly* *↑*	100/0/0
Left Rib BMC (g)	87.8 ± 22.9	91.2 ± 16.4	6.8 ± 13.3	0.14 ± 0.37	*Unclear*	39/55/6
Left Rib BMD (g∙cm^−2^)	0.62 ± 0.07	0.69 ± 0.07	11.6 ± 3.7	0.93 ± 0.31	*Almost Certainly* *↑*	100/0/0
Right Rib BMC (g)	86.5 ± 23.3	88.7 ± 13.6	5.9 ± 12.2	0.09 ± 0.32	*Unclear*	28/66/6
Right Rib BMD (g∙cm^−2^)	0.62 ± 0.06	0.67 ± 0.06	8.4 ± 2.9	0.83 ± 0.29	*Almost Certainly* *↑*	100/0/0

FFM = fat-free mass; BMC = bone mineral content; BMD = bone mineral density; CL = confidence limits.
